# Supplementary material for: Fatty acids synergize with bacteriophages against multidrug-resistant pathogens
Source: bioRxiv. 2026 Oct 28:2026.10.27.759484. Preprint. [Version 1] doi: 10.64898/2026.10.27.759484 (PMC13733972; doi:10.64898/2026.10.27.759484)
Supplement: Supplementary file 2 [file media-2.pdf]

Fatty acids synergize with bacteriophages against multidrug-resistant pathogens

Kayla R. Raygoza, Andrew Saghir, Yu Chen, Faith Cribbs, Ritwik Kumar, Liana Hase-Penn, Kyle Kim, Alisha Monsibais, Gina Faraci, Katrine L. Whiteson*, Sage J. B. Dunham*

Department of Molecular Biology and Biochemistry, University of California, Irvine, California, USA.

*Co-corresponding authors:
Katrine L. Whiteson: 3315 McGaugh Hall, Irvine, CA, 92697; katrine@uci.edu; 949-824-3509
Sage J. B. Dunham: 3315 McGaugh Hall, Irvine, CA, 92697; sjdunham@uci.edu; 949-824-3509

Supplemental Information

Supplemental Table 1: List of strains.
Supplemental Table 2: List of phages.
Supplemental Table 3: List of key reagents.

Supplemental Table 1: List of strains.

Species	Strain	Source	Isolated from:	abx resistance profile
Stenotrophomonas maltophilia	K279a	STM type Strain	NCBI #ASM7248v1	unknown
Stenotrophomonas maltophilia	B28B	UCSD	blood infection	unknown
Stenotrophomonas maltophilia	FL10	UCI	Florida, Pulmonary infection	unknown
Stenotrophomonas maltophilia	Lipton23	UCI	Pulmonary infection	unknown
Stenotrophomonas maltophilia	HLP	UCI	Pulmonary infection	unknown
Stenotrophomonas maltophilia	SM10KA	UCSD	Lesio MTAE8 CXBS	unknown
Stenotrophomonas maltophilia	SM12LS	UCSD	UR MTAE8 CXURN	unknown
Stenotrophomonas maltophilia	SM15KA	UCSD	BRONW MTAE8 CXQBR	unknown
Stenotrophomonas maltophilia	SM16LS	UCSD	SITE MAC-S CXBS	unknown
Stenotrophomonas maltophilia	SM20TB	UCSD	UR MACU CXURN	ceftazadime, levofloxacin
Stenotrophomonas maltophilia	SM25LS	UCSD	UR MAC-S CXURN	unknown
Stenotrophomonas maltophilia	SM26KA	UCSD	BRONW MAC CXRES	unknown
Stenotrophomonas maltophilia	SM27KA	UCSD	UR MTAE8 CXURN	ceftazadime
Stenotrophomonas maltophilia	SM32KA	UCSD	BRONW CNC-8 CXAFB	unknown
Stenotrophomonas maltophilia	SM49LS	UCSD	RESCF MTAE8 CXRES	levofloxacin
Stenotrophomonas maltophilia	SM50JS	UCSD	Rescf MTAE8 CXRES	resistant to all
Stenotrophomonas maltophilia	SM73PII	UCSD	CXRES	ceftazadime, levofloxacin
Stenotrophomonas maltophilia	SM74PII	UCSD	UR MAC-S CXURN	unknown
Staphylococcus aureus	10,6	UCI - Whiteson Lab Wound Collection	Left thumb (abscess)	unknown
Staphylococcus aureus	12,21	UCI - Whiteson Lab Wound Collection	Right femur	unknown
Staphylococcus aureus	2,4	UCI - Whiteson Lab Wound Collection	Left chin (abscess)	unknown
Staphylococcus aureus	27,51	UCI - Whiteson Lab Wound Collection	Sinus	unknown

Staphylococcus aureus	30,59	UCI - Whiteson Lab Wound Collection	Scalp skin	unknown
Staphylococcus aureus	32,63	UCI - Whiteson Lab Wound Collection	Right ankle (drainage)	unknown
Staphylococcus aureus	19,36	UCI - Whiteson Lab Wound Collection	Left hand (skin lesion)	unknown
Staphylococcus aureus	27,52	UCI - Whiteson Lab Wound Collection	Sinus	unknown
Staphylococcus aureus	9,15	UCI - Whiteson Lab Wound Collection	Right eyebrow (abscess)	unknown
Staphylococcus aureus	10,18	UCI - Whiteson Lab Wound Collection	Left thumb (abscess)	unknown
Staphylococcus aureus	21,40	UCI - Whiteson Lab Wound Collection	Lower left leg (drainage)	unknown
Staphylococcus aureus	25,46	UCI - Whiteson Lab Wound Collection	Right arm (skin; drainage)	unknown
Staphylococcus aureus	7,12	UCI - Whiteson Lab Wound Collection	Abdomen (wound)	unknown
Staphylococcus aureus	JVS2311	UCI Medical Center, Donald Forthal	MRSA; external medical device	unknown
Staphylococcus aureus	G565 ATCC 33591	Greg Burkeen, FLR, SDSU	?	unknown
Staphylococcus aureus	31,60	UCI - Whiteson Lab Wound Collection	Lower left leg (lesion)	unknown
Staphylococcus aureus	PedCF	Greg Burkeen, FLR, SDSU		unknown
Staphylococcus aureus	USA300	UCI - Whiteson Lab Wound Collection	ATCC BAA-1680	unknown
Pseudomonas aeruginosa	4,7	UCI - Whiteson Lab Wound Collection	Sinus (chronic sinusitis)	unknown
Pseudomonas aeruginosa	12,22	UCI - Whiteson Lab Wound Collection	Right femur	unknown
Pseudomonas aeruginosa	14,26	UCI - Whiteson Lab Wound Collection	Left ear	unknown
Pseudomonas aeruginosa	16,30	UCI - Whiteson Lab Wound Collection	Sinus	unknown
Pseudomonas aeruginosa	20,38	UCI - Whiteson Lab Wound Collection	Nose (wound)	unknown
Pseudomonas aeruginosa	24,44	UCI - Whiteson Lab Wound Collection	Sinus	unknown
Pseudomonas aeruginosa	26,48	UCI - Whiteson Lab Wound Collection	Left thigh	unknown
Pseudomonas aeruginosa	PAnmFLR01 (P1)	KW (CF isolate UCSD 2014)	unknown	unknown
Pseudomonas aeruginosa	WT P14	Siryaporn (O'Toole)	unknown	unknown

Pseudomonas aeruginosa	P14 DPhz	Hochbaum / Newman	unknown	unknown
Pseudomonas aeruginosa	11,19	UCI - Whiteson Lab Wound Collection	Sputum	unknown
Pseudomonas aeruginosa	17,32	UCI - Whiteson Lab Wound Collection	Sinus	unknown
Pseudomonas aeruginosa	ESKAPE Pa	ESKAPE Pathogens	unknown	unknown
Pseudomonas aeruginosa	PAO1	Dorrestein/Phelan	unknown	unknown
Enterococcus faecalis	EF01PII	David Pride	bone left foot	none
Enterococcus faecalis	EF02PII	David Pride	urine	none
Enterococcus faecalis	EF03PII	David Pride	urine	gentamicin-syn
Enterococcus faecium	EF04PII	David Pride	blood	amp, penicillin G
Enterococcus faecium	EF05PII	David Pride	urine	amp, penicillin G, vancomycin
Enterococcus faecium	EF06PII	David Pride	blood picc line	amp, penicillin G, tetracycline, vanco
Enterococcus faecalis	EF07PII	David Pride	urine	gentamicin-syn
Enterococcus faecalis	EF08PII	David Pride	blood	none
Enterococcus faecalis	EF09PII	David Pride	urine	tetracycline, vanco
Enterococcus faecalis	EF10PII	David Pride	body site lesion leg	none
Enterococcus faecium	EF11PII	David Pride	body site pancreatic fluid	linezolid
Enterococcus faecalis	EF12PII	David Pride	bile duct fluid	none
Enterococcus faecalis	EF13PII	David Pride	body site abscess	none
Enterococcus faecalis	EF14PII	David Pride	urine	gentamicin-syn
Enterococcus faecalis	EF15PII	David Pride	urine	none
Enterococcus faecium	EF16PII	David Pride	urine	none
Enterococcus faecalis	EF17PII	David Pride	urine	none
Enterococcus faecium	EF18PII	David Pride	body site pancreas	amp, penicillin G, tetracycline, vanco
Enterococcus faecalis	EF19PII	David Pride	urine	none
Enterococcus faecium	EF20PII	David Pride	body fluid wound	amp, penicillin G, tetracycline, vanco
Enterococcus faecalis	EF23PII	David Pride	urine	gentamicin-syn
Enterococcus faecalis	EF24PII	David Pride	abscess	none
Enterococcus faecalis	EF25PII	David Pride	blood	none
Enterococcus faecalis	EF28PII	David Pride	urine	none
Enterococcus faecalis	EF29PII	David Pride	urine	none
Enterococcus faecalis	EF30PII	David Pride	urine	none
Enterococcus faecalis	EF31PII	David Pride	urine	none

Enterococcus faecalis	EF32PII	David Pride	urine	none
Enterococcus faecalis	EF33PII	David Pride	urine	none
Enterococcus faecalis	EF35PII	David Pride	urine	none
Enterococcus faecalis	EF36PII	David Pride	urine	none
Enterococcus faecalis	EF37PII	David Pride	urine	gentamicin-syn
Enterococcus faecalis	EF38PII	David Pride	urine	gentamicin-syn
Enterococcus faecalis	EF39PII	David Pride	bile duct fluid	none
Enterococcus faecalis	EF40PII	David Pride	urine	none
Enterococcus faecium	EF41PII	David Pride	body site swab	amp, gentamicin-syn, penicillin G, tetra, vanco
Enterococcus faecalis	EF42PII	David Pride	body site abdomen	gentamicin-syn
Enterococcus faecalis	EF43PII	David Pride	urine	none
Enterococcus faecalis	EF44PII	David Pride	urine	none
Enterococcus faecalis	EF45PII	David Pride	urine	none
Enterococcus faecalis	EF46PII	David Pride	urine	none
Enterococcus faecalis	EF47PII	David Pride	urine	gentamicin-syn
Enterococcus faecium	EF48PII	David Pride	urine	amp, penicillin G, tetracycline, vanco
Enterococcus faecalis	EF49PII	David Pride	urine	gentamicin-syn
Enterococcus faecium	EF50PII	David Pride	urine	amp, penicillin G, vanco
Enterococcus faecalis	EF51PII	David Pride	urine	none
Enterococcus faecalis	EF52PII	David Pride	urine	none
Enterococcus faecium	EF53PI	David Pride	aspirate abdominal fluid	none
Enterococcus faecalis	EF54PII	David Pride	aspirate abdominal fluid	none
Enterococcus faecalis	EF55PII	David Pride	urine	none
Enterococcus faecalis	EF57PII	David Pride	drainage ear	none
Enterococcus faecalis	EF58PII	David Pride	aortic abscess	gentamicin-syn
Enterococcus faecalis	EF59PII	David Pride	urine	none
Enterococcus faecalis	EF61PII	David Pride	urine	gentamicin-syn
Enterococcus faecium	EF62PII	David Pride	body tissue ulcer	amp, penicillin G, tetra, vanco
Enterococcus faecalis	EF63PII	David Pride	body site mouth swab	unknown
Enterococcus faecalis	EF64PII	David Pride	urine	none
Enterococcus faecalis	EF65PII	David Pride	urine	none
Enterococcus faecalis	EF66PII	David Pride	urine	none
Enterococcus faecalis	EF67PII	David Pride	urine	gentamicin-syn
Enterococcus faecalis	EF68PII	David Pride	urine	none
Enterococcus faecalis	EF69PII	David Pride	urine	gentamicin-syn
Enterococcus faecalis	EF70PII	David Pride	urine	gentamicin-syn

Enterococcus faecalis	EF71PII	David Pride	urine	none
Enterococcus faecalis	EF72PII	David Pride	urine	none
Enterococcus faecalis	EF73PII	David Pride	urine	none
Enterococcus faecalis	EF74PII	David Pride	peritoneal fluid	minocycline, gentamycin-syn
Enterococcus faecalis	EF75PII	David Pride	aspirate abdominal fluid	none
Enterococcus faecalis	EF76PII	David Pride	aspirate abdominal fluid	none
Enterococcus faecalis	EF77PII	David Pride	urine	none
Enterococcus faecalis	EF78PII	David Pride	unknown	unknown
Enterococcus faecium	EF79PII	David Pride	body site swab	amp, penicillin G, tetra, vanco
Enterococcus faecalis	EF80PII	David Pride	unknown	unknown
Enterococcus faecalis	EF81PII	David Pride	body tissue finer	none
Enterococcus faecalis	EF82PII	David Pride	urine	none
Enterococcus faecalis	EF83PII	David Pride	urine	none
Enterococcus faecalis	EF84PII	David Pride	urine	gentamycin-syn
Enterococcus faecium	EF85PII	David Pride	blood	amp, penicllin G
Enterococcus faecalis	EF86PII	David Pride	urine	none
Enterococcus faecalis	EF87PII	David Pride	urine	none
Enterococcus faecalis	EF88PII	David Pride	urine	none
Enterococcus faecium	DP17	David Pride	unknown	unknown
Enterococcus faecalis	Yi6	BEI	unknown	unknown
Enterococcus faecium	TX1330	BEI	unknown	unknown
Enterococcus faecalis	V587	BEI	unknown	unknown
Enterococcus faecalis	DP05	David Pride	unknown	unknown
Enterococcus faecalis	DP12	David Pride	unknown	unknown
Enterococcus faecalis	DP15	David Pride	unknown	unknown
Enterococcus faecalis	EF102PII	David Pride	unknown	unknown
Enterococcus faecalis	EF116PII	David Pride	unknown	unknown
Enterococcus faecalis	ESKAPE E. faecalis	ESKAPE Pathogens	unknown	unknown

Supplemental Table 2: List of phages.

Host organism	Phage name	Host strain	Isolation Source	Isolated By	Morphology	Genome Ref	Citation
S. maltophilia	ANB28	B28B	Escondido Wastewater	Pride Lab-Alisha Blanc	Podovirus	PQ434692	Monsibais 2025
S. maltophilia	KB824	B28B	Escondido Wastewater	Pride Lab	Siphovirus	PQ434693	Monsibais 2025
S. maltophilia	SPB2Φ2	B28B	Escondido Wastewater	Pride Lab	Podovirus	PQ074228	Monsibais 2025
S. maltophilia	ESCSMPI	SM71PII	Escondido Wastewater	OCPT- Coby Fan	Siphovirus		
E. faecalis	CAR	Yi6	Escondido Wastewater	OCPT-Stephen Wandro	Myovirus		
S. aureus	Ludmila	USA300	SCCWRP wastewater	OCPT-Alexandra Albert	Podovirus		
P. aeruginosa	PAPS1	ESKAPE PA	Escondido Wastewater	OCPT-Ritwik Kumar	Unknown		

Supplemental Table 3: List of key reagents.

Type	Supplier	Compound	Product number
Acid	Sigma	Formic acid	695076-100ML
Acid	Fluka	Acetic acid	49199-50ML-F
Acid	Fisher	Propionic acid	S25508
Acid	VWR	Butyric acid	TCB0754-25ML (EA)
Acid	VWR	Pentanoic acid	TCV0003-25ML (EA)
Acid	VWR	Hexanoic acid	TCH0105-25ML (EA)
Acid	VWR	Heptanoic acid	TCH0030-25ML (EA)
Acid	VWR	Octanoic acid	TCO0027-25ML (EA)
Acid	VWR	Nonanoic acid	TCP0952-25ML (EA)
Acid	Fisher	Decanoic acid	D001725G
Base	Sigma	Sodium formate	247596-100G
Base	Sigma	Sodium acetate	S2889-250G
Base	Sigma	Sodium propionate	P1880-100G
Base	Sigma	Sodium butyrate	303410-5G
Base	Ambeed, Inc.	Sodium pentanoate	AMBH2D6FA579-25G
Base	Sigma	Sodium hexanoate	C4026-10G
Base	Ambeed, Inc.	Sodium heptanoate	AMBH9614F4B7-25G
Base	Sigma	Sodium octanoate	C5038-10G
Base	Sant Cruz Biotech	Sodium nonanoate	296399
Base	Sigma	Sodium decanoate	C4151-5G
Antibiotic	Thomas Scientific	Meropenam	C817F47 (EA/1)
Antibiotic	Sigma-Aldrich	Cotrimoxazole (SXT)	A2487-1ML
Antibiotic	Thomas Scientific	Levofloxacin	C790J97
Antibiotic	Thomas Scientific	Minocyclin	C828G93
Antibiotic	Neta Scientific	Ceftazidime Hydrate	RPI-C59200-1.0
Antibiotic	Fisher	Polymixin B Sulfate	AAJ671638EQ
